# Physical exercise associated with improved BMD independently of sex and vitamin D levels in young adults

**DOI:** 10.1007/s00421-016-3383-1

**Published:** 2016-05-05

**Authors:** Rune Tønnesen, Peter Schwarz, Peter Hambak Hovind, Lars Thorbjørn Jensen

**Affiliations:** Department of Clinical Physiology and Nuclear Medicine, Rigshospitalet, Nordre Ringvej 57, Glostrup, 2600 Copenhagen, Denmark; Department of Endocrinology PE and Research Centre of Ageing and Osteoporosis, Rigshospitalet, Copenhagen, Denmark; Department of Clinical Physiology and Nuclear Medicine, University Hospital of Herlev, Copenhagen, Denmark; Faculty of Health Sciences, University of Copenhagen, Copenhagen, Denmark

**Keywords:** Vitamin D, Young adults, Sex, Physical exercise, Bone, BMD

## Abstract

**Purpose:**

Young men and women accrue the majority of their bone mass in their teens and twenties, where their bone mass peaks (PBM), yet little is known about the roles of physical exercise, vitamin D levels and bone mineral density (BMD) near PBM.

**Methods:**

To comparatively examine the effect of physical exercise and two vitamin D levels (insufficient s-25[OH]D <50 nmol/L and sufficient s-25[OH]D >80 nmol/L) on the BMD measured at the femoral neck, total hip (bilaterally) and the lumbar spine (L2–L4) in male and female participants approaching PBM.

**Results:**

The insufficient s-25[OH]D group, median age 21.6 (19.8–22.8) years, and BMI 24.2 ± 5.0 kg/m^2^ had BMD 0.10 (0.03, 0.17) g/cm^2^ (*p* = 0.008) lower at all DXA-scan sites compared to the sufficient s-25[OH]D group, median age 19.5 (19.0–22.3) years, and BMI of 22.6 ± 1.8 kg/m^2^. Exercise was positively associated with the BMD at all DXA-scan sites (*p*_trend_ = 0.0001) and with equal benefit; there was no interaction between exercise and the DXA-scan site (*p* = 0.09). The male participants did not have a systematically higher BMD than the female participants for all scan sites; only for hips total and femoral neck bilaterally, while it was equal at the lumbar spine.

**Conclusion:**

The BMD in young healthy adults is associated with physical exercise, independent of sex and s-25[OH]D status. A sufficient s-25[OH]D status was systematically associated with a higher BMD for all levels of exercise. For both sexes and vitamin D levels exercise was equally positively associated with BMD.

## Introduction

Long term vitamin D deficiency is a well-known cause of osteomalacia and rickets. Nevertheless, while the prevention of rickets is often addressed, little attention is paid to the vitamin D status in late adolescents. Human bones accrue mass from the fetal state until they reach a peak bone mass (PBM). This process is finalized for the hips roughly at the ages of 18–20 years and at the lumbar spine at the ages of 20–25 years (Lu et al. 2014). In childhood and early adulthood, physical exercise can increase the accrual of bone mass (Meyer et al. 2011; Eleftheriou et al. 2012; Kemmler et al. 2015; Stagi et al. 2013). After the peak bone mass (PBM) is reached, however, there is a loss of BMD at the hips and spine throughout the life span (Matkovic et al. 1994); for women, the loss of bone mass accelerates after menopause (Finkelstein et al. 2008). PBM is thus a vital consideration in the pathogenesis of osteoporosis. The peak bone mass and the bone loss due to aging are the two major factors that determine the onset of osteoporosis (Hernandez et al. 2003) and factor into the risk of future osteoporotic fractures.

Human vitamin D is stored as 25-hydroxyvitamin D (25[OH]D); when its levels decrease, the parathyroid hormone (PTH) secretion increases, and the higher PTH concentration, among other signaling cascades, stimulates osteoclastic bone resorption. The interrelationship between serum (s-) 25[OH]D and s-PTH has been utilized in the classification of vitamin D levels (Lips 2004). Notably, the optimal vitamin D level is still debated. Vitamin D insufficiency, defined as s-25[OH]D <50 nmol/L, has been found to be associated with an increased risk of mortality, cancer, infections, and both cardiovascular and metabolic diseases (Durup et al. 2012, 2015; Autier et al. 2014; Aregbesola et al. 2013; Cheng et al. 2010). Ideally, the target for a beneficial vitamin D status should achieve the maximal positive and minimal negative effects; the concentration range of 50–80 nmol/L of s-25[OH]D appears to achieve this goal, as mortality is at its lowest and the s-PTH is not elevated within this range (Durup et al. 2012; Lips 2004; Bischoff-Ferrari 2014).

The biologically active form of vitamin D, or 1,25-dihydroxyvitamin D (1,25[OH]D), stimulates the absorption of calcium (Christakos et al. 2011) and phosphate (Lederer 2014) from the intestine, and it increases the renal absorption of calcium. There is a positive association between increases in exercise and increases in calcium absorption (Teerapornpuntakit et al. 2009). The hormonal structure of 1,25[OH]D is involved in the elongation, remodeling and calcification of bones (Lui et al. 2010; Boyan et al. 2003; Dusso et al. 2005). In the bone remodeling process, the skeletal system is renewed and maintained by a series of different hormones, i.e., estrogen, PTH, prostaglandins, 1,25[OH]D, and other factors (Bonewald 2011; Charles and Aliprantis 2014).

In addition to their mineral content, the shape of the bones also contributes to their strength. The shape of bones is a result of adaptation to external stimuli or a lack of thereof (Skerry 2008). The two primary types of external loads are torsion and bending, and these forces lead to different bone adaptation responses. In general, torsion leads to an increased circumference and a bending into oval elongation (van der Meulen et al. 2001). The application of a mechanical load to the bone induces an adaptation in the bone (Duncan and Turner 1995) that leads to bone remodeling (Bonewald 2011). The increased bone strength is a result of the structural adaptation to the increased mechanical load on the bone (Skerry 2008).

Weight-bearing training such as tennis (Ermin et al. 2012), high-impact aerobics (Liang et al. 2011), jogging (high activity) (Deere 2012), stair climbing (Gianoudis et al. 2014), and muscle–strengthening exercises such as squatting (Mosti et al. 2014) and resistance training (Borba-Pinheiro et al. 2016; Lesinski et al. 2016) are important for building and maintaining bone density. Endurance exercise types such as swimming, cycling and long-distance running have no positive effect on BMD (Scofield and Hecht 2012); however, dancing is disputed (Amorim et al. 2015).

The resulting mineralization is manifested by the bone mineral density (BMD). Bone scanning by dual X-ray absorptiometry (DXA) is routinely used to assess the areal bone mineral density (BMD), both in clinical (Cummings et al. 2002) as well as in investigative settings to provide information regarding the bone status. While Vitamin D and calcium are essential for the calcification of the bone tissue, the stimulation through physical exercise is important as well. Traditionally, the effect of exercise on bone is examined using multiple analyses of single-site to single-site comparisons, i.e., left hip-to-neck to right hip-to-neck. Nevertheless, very little is known about the influence of vitamin D levels and exercise on bone mineralization in young adults at an age close to the PBM by utilizing a direct comparison between sites of the bone DXA. In this study, we comparatively examine the effects of physical exercise and s-25[OH]D level on the BMD measured at the femoral neck, total hip (bilaterally), and the lumbar spine (L2–L4).

## Methods

### Design and participants

The study was carried out from August 2012 to December 2013 as a cross-sectional investigation and was conducted at the Department of Clinical Physiology, Nuclear Medicine and PET, Rigshospitalet Glostrup.

The study was comprised of 29 young male adults with a mean age of 21.7 ± 2.6 and 68 young female adults with a mean age of 20.4 ± 2.1. The study population is a subsample (*n* = 97) of a large cohort (*n* = 698) of young adults aged 18–25 years who were screened for vitamin D status (data not yet published). From the main screenings cohort, we then recruited two groups. The first group (*n* = 36) was composed of individuals known to be vitamin D sufficient (s-25[OH]D >80 nmol/L) and was thus referred to as the vitamin D sufficient group. The second group (*n* = 61) had insufficient vitamin D concentration (s-25[OH]D <50 nmol/L) and was referred to as the vitamin D insufficient group. Only subjects who did not take supplementation were included in the study; thus, each subject’s respective vitamin D level was a result of their dietary intake, behavior and genetics.

An invitation was sent by e-mail to 314 individuals with s-25[OH]D insufficiency. A total of 61 accepted the invitation (19.4 %). An invitation was also sent to 90 individuals with s-25[OH]D sufficiency, and 36 accepted the invitation (40.0 %). This study was conducted in accordance with the Helsinki declaration, and all participants gave their informed written consent before their inclusion. The study was additionally approved by the regional ethics committee (Ethics Committee for Region Hovedstaden) ref. no. H-1-2012-023.

### Measurement

Through the use of a questionnaire, the participants habits of exercise, smoking and alcohol consumption were obtained. For smoking, “How many cigarettes have you smoked in the last 7 days?,” with responses grouped into smoking yes/no. For alcohol, “How many units (12 g) of alcohol have you drank in the last 7 days?,” with responses grouped into alcohol yes/no. Lastly, for exercise habits “How many hours have you spent on exercise in the last 7 days?” was grouped into responses of 0–1/2, 1/2–2, 2–4, 4–7, or 7 or more.

The medical history for each participant was obtained through a personal interview to ensure that the subjects were clinically healthy. It was ensured that no participants had any diseases or took any vitamin D supplementation or medications affecting bone metabolism.

We drew a venous non-fasting blood sample of 20 mL for the measurement of s-PTH, s-ionized calcium, s-creatinine and remeasurement of s-25[OH]D. Within an hour of sampling, the routine local laboratory performed the s-25[OH]D analysis. The participant’s heights were measured using a vertically mounted cm ruler. Furthermore, the participant’s weights were measured using an electronic scale (OBH Nordic 6295). The body mass index (BMI) was calculated as the measured weight in kg divided by the measured height in meters-squared.

### Dual X-ray absorptiometry parameters

The BMD was analyzed by use of a dual X-ray Absorptiometry (DXA) scanner (Lunar Prodigy Advance, GE Healthcare, Madison, WI, enCORE ver. 14.10). We used routine measurements of the BMD at the total hip, the femoral neck (bilaterally), and at the lumbar spine (L_2_–L_4_). To detect and correct for any drift, a standard quality assurance and stability monitoring of the DXA scanner with the GE Lunar calibration block was performed daily before all scanning procedures. The same scanner was used for all scans, and the same trained operator performed all scans and analyses. The intra-scanner coefficient of variation (CV) was 0.27 %. BMD are reported as the mean with 95 % confidence limits (Cl).

### Laboratory analysis

#### Serum-25-hydroxyvitamin D

The serum-25[OH]D was analyzed by utilizing chemiluminescent immunoassay technology [Liaison^®^ 25-OH Vitamin D Total Assay; Diasorin Inc., Saluggia (Vercelli), Italy]. This test does not differentiate between the s-25[OH]D metabolites D_2_ and D_3,_ and the data are thus presented as the s-25[OH]D_2+3_. The inter-assay CVs were 9.8 % at a mean value 39.0 nmol/L and 10.0 % at a mean value of 136.0 nmol/L. The intra-assay CV was 2.5 %.

#### Serum-creatinine, serum intact parathyroid hormone, and s-ionized-calcium

The biochemical variables were analyzed by use of commercial kits or standard laboratory methods.

The serum intact parathyroid hormone (s-iPTH) (VITROS^®^ Intact PTH Ortho-Clinical Diagnostics, Rochester, NY, USA,) was analyzed using the VITROS 5600 system, inter-assay and intra-assay CV < 7.6 % (Tan et al. 2013).

The serum-Creatinine (s-Cr) was analyzed with the Vitros 5600 system (VITROS^®^ CREA-slides Ortho-Clinical Diagnostics, Rochester, NY, USA), inter- and intra-assay CV < 5 %.

The serum-Ionized-Calcium (s-Ca)was analyzed, utilizing Nova 8 (Bowers et al. 1986), (NOVA Biomedical, Waltham, USA, inter- and intra-assay CV < 1.1 %).

### Statistical analysis

Given that the least clinically relevant difference for the BMD in the lumbar spine is 0.1 g/cm^2^, the study required at least 48 participants in each group to achieve a standard deviation (SD) of 0.14 g/cm^2^, an alpha of 0.05 and a power of 0.80. We rounded up to 50 participants, as we expected a 4 % drop-out. The continuous data were reported as the means and SDs when the data followed a normal distribution, and they were reported as medians and interquartile range (IQR) when they did not. Visual inspections of the continuous data were used to determine the normality of the distribution. For the categorical data, percentages were used to assess the distribution. We selected a *p* value of *p* < 0.05 to be statistically significant. Adjusted results are reported as mean (95 % CI).

To determine if subjects had crossed-over from one group to another in the time between screening and scan, boundaries were calculated as limit ± 2 CV limit. The upper boundary for insufficient group with an upper limit of 50 nmol/L and CV of 9.8 % was calculated to 60 nmol/L. The lower boundary for the sufficient group with lower limit of 80 nmol/L and CV of 9.8 % to 64 nmol/L.

Mixed model utilizing repeated measures was used for the analysis of the BMD, assuming “vitamin D level,” “exercise” and “sex” as fixed factors. The mixed model approach has advantages over multiple ANCOVAs, as it allows for more generalizable interpretations. Namely, the results from a mixed model represent random subjects and can take into account the within- and between-subject biological variation. A Restricted Maximum Likelihood (REML) was applied as the estimation method. The covariance was unstructured. Lastly, all statistical analyses were performed using SAS statistical software (SAS^®^ Version 9.3, Cary, NC, USA).

## Results

### Physical characteristics of subjects

The baseline physical characteristics of the participants are presented in Table 1. We observed no significant differences in the height and weight between the two groups of participants. At screening the sufficient group had a mean s-25[OH]D of 106.9 ± 23.5 nmol/L, the time from screening (median 95 % CI) was 16 (9, 61) days and at scan mean s-25[OH]D was 90.0 ± 23.8 nmol/L and no participant had dropped below the boundary. At screening the insufficient group had a mean s-25[OH]D of 31.7 ± 11.3 nmol/L, the time from screening (median 95 % CI) was 29 (12, 54) days and at scan mean s-25[OH]D was 34.9 ± 19.2 nmol/L and five participants were above the boundary. However, the participants with an insufficient vitamin D level had a higher BMI and s-iPTH level, as well as a lower level of s-Cr, albeit all values fell within the normal physiological parameters. Furthermore, we found that the participants with a higher vitamin D level consumed more alcohol on a weekly basis.

**Table 1:** Characteristics of subjects, by vitamin D level

Variable	Sufficient (*N* = 36)	Insufficient (*N* = 61)
Age (years)	19.5 (19.0, 22.3)	21.6 (19.8, 22.8)
Height (m)	173.6 ± 9.3	170.6 ± 8.5
Body mass index	22.6 ± 1.8	24.2 ± 5.0
Days from screening to scan	16 (9, 61)	29 (12, 54)
S-25[OH]D screening (nmol/l)	106.9 ± 23.5	31.7 ± 11.3
S-25[OH]D reassessment (nmol/l)	90.0 ± 23.8	34.9 ± 19.2
PTH	37 (31, 41)	50 (14, 88)
Creatine (µmol/L)	72.4 ± 12.0	66.3 ± 14.4
Calcium (pmol/L)	1.25 ± 0.04	1.26 ± 0.04
Sex		
Women	26 (72.2 %)	42 (68.9 %)
Men	10 (27.8 %)	19 (31.1 %)
Exercise/hours (last 7 days)		
0–1/2	3 (8.3 %)	19 (31.1 %)
1/2–4	10 (27.8 %)	30 (49.2 %)
4–7	4 (11.1 %)	4 (6.6 %)
7+	19 (52.8 %)	8 (13.1 %)
Tobacco (last 7 days)		
Missing	1 (2.8 %)	1 (1.6 %)
No	27 (75.0 %)	42 (68.9 %)
Yes	8 (22.2 %)	18 (29.5 %)
Alcohol (last 7 days)		
No	11 (30.6 %)	35 (57.4 %)
Yes	25 (69.4 %)	26 (42.6 %)

Normal distributed values are presented as the mean ± SD, skewed distributions are presented as Median (95 % CI)

### Measurements of bone mineral density

In our study, we found a positive association between exercise and the BMD at all DXA-scan sites (*p*_trend_ = 0.0001). At each DXA-scan site, we observed an equal benefit of exercise, as there was no interaction between exercise and the DXA-scan site (*p* = 0.09). The adjusted BMD for the highest exercise level of +7 h/week was significantly higher when compared with all of the other levels of exercise. The BMD was 0.142 (0.059, 0.225) g/cm^2^ (*p* = 0.001), 0.140 (0.070, 0.210) g/cm^2^ (*p* = 0.0002), and 0.134(0.0290, 0.240) g/cm^2^ higher (*p* = 0.01) for the 0–1/2, 1/2–4, and 4–7 h/week exercise levels, respectively. When comparing between the exercise levels of 0–1/2, 1/2–4, and 4–7 h/week, the adjusted BMD was not significantly different.

**Fig. 1:**
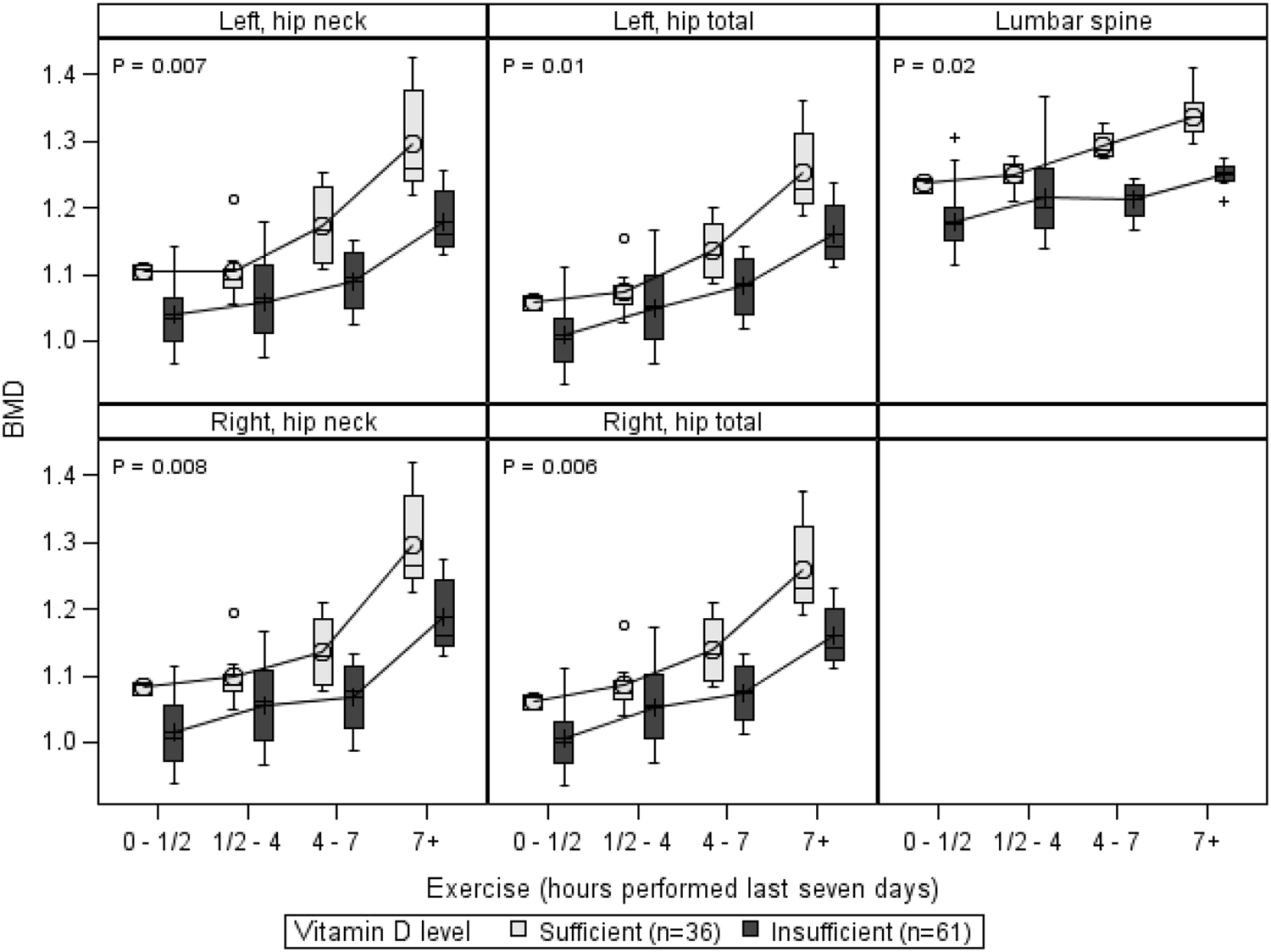
Vitamin D levels—BMD (g/cm^2^) and exercise (hours performed over last 7 days) by scan site, adjusted for BMI (kg/m^2^). *p* values are from main effects for vitamin D levels compared at each scan site. The *box*, interquartile range; *horizontal line* inside the *box*, median value; *symbol* (*cross or circle*), mean value; T-shaped caps, max. and min. values

The vitamin D insufficient group had a systematically lower BMD 0.10 ± 0.03 g/cm^2^ (*p* = 0.008) at all DXA-scan sites when compared to the vitamin D sufficient group, see Fig. 1. The observed increase in the BMD as the exercise level increased was the same for both vitamin D sufficient and insufficient groups; furthermore there was no interaction between exercise and S-25[OH]D level (*p* = 0.3).

**Fig. 2:**
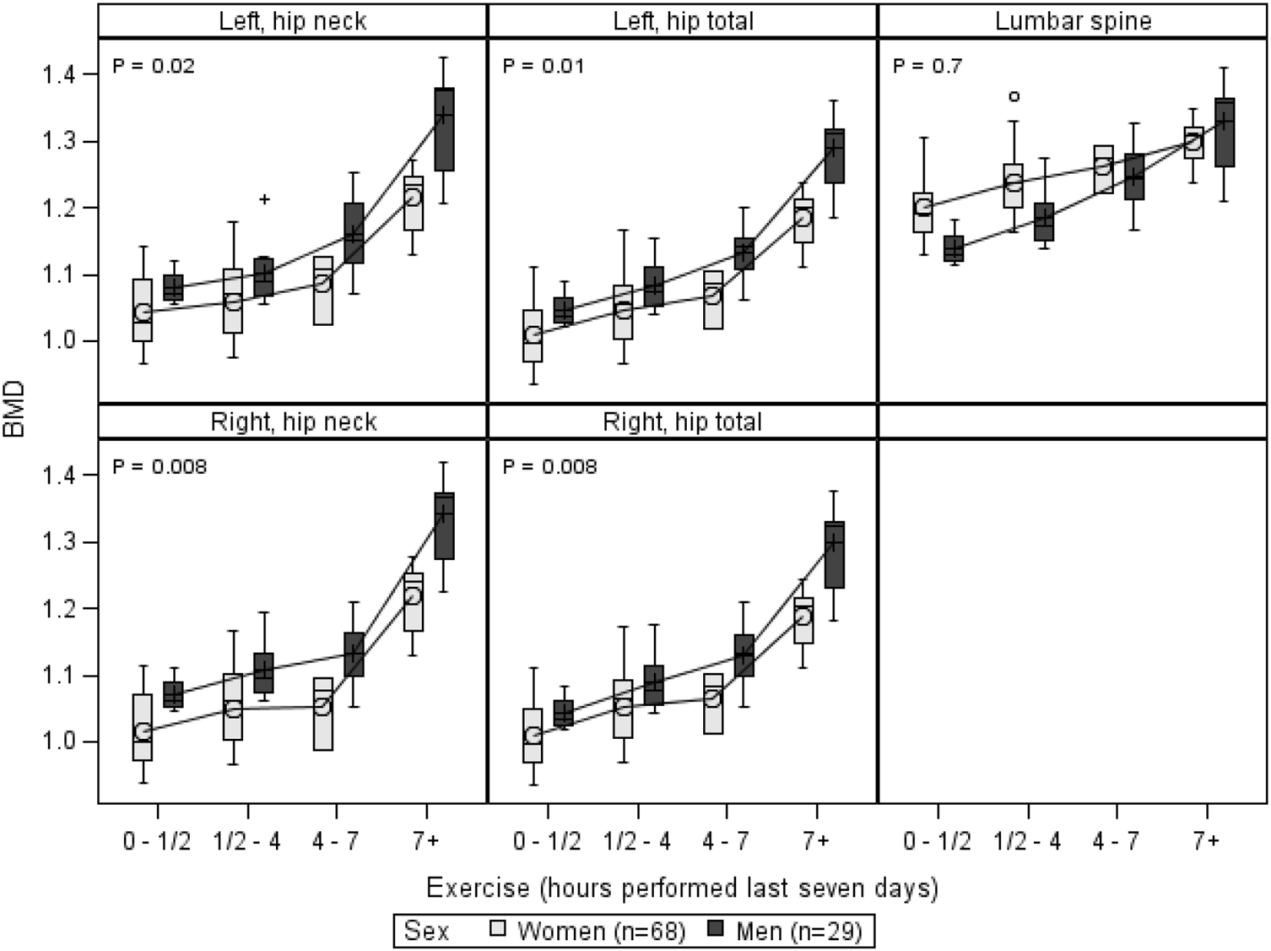
Sex—BMD (g/cm^2^) and exercise (hours performed last 7 days) by scan site, adjusted for BMI (kg/m^2^). *p* values are from main effects for vitamin D levels compared at each scan site. The *box*, interquartile range; *horizontal line* inside the *box*, median value; *symbol* (*cross or circle*), mean value; T-shaped caps, max. and min. values

Furthermore, we observed that there was an interaction between the variables of sex and DXA-scan site (*p* = 0.002), as the male participants did not have a higher BMD than the females for all scan sites, see Fig. 2. For this reason, there may be differentiating effect of sex at certain DXA-scan sites, while this sex effect may absent at others. The males had a higher BMD than the women, with differences as follows: at the total left hip 0.076 (0.015, 0.136) g/cm^2^ (*p* = 0.02), left hip neck 0.084 (0.012, 0.16) g/cm^2^ (*p* = 0.02), total right hip 0.076 (0.016, 0.135) g/cm^2^ (*p* = 0.01) and right hip neck 0.09249 (0.022, 0.163) g/cm^2^ (*p* = 0.01). This was not preserved in the lumbar the spine, where the difference between male and female BMD was −0.011 (−0.073, 0.050) g/cm^2^ (*p* = 0.72). The effect of sex on the BMD was only present at the total hip and femoral neck, bilaterally, while it was absent in the lumbar spine.

Both the male and female participants demonstrated an equal effect of exercise. The increase in the BMD as an effect of exercise was the same for both sexes, and there was no interaction between gender and exercise (*p* = 0.4).

The s-Cr, s-iPTH and s-Ca were all within the ranges of normal serum concentrations and had no significant impact on the BMD. As a result, those data were excluded from the final model. We found that an increase in the single BMI unit increased the BMD by 0.010 (0.004, 0.016) g/cm^2^ (*p* = 0.002).

Lastly, the smoking or alcohol intake of the subjects did not affect the BMD at any site for either gender in our study and, thus, was not included in the final model.

## Discussion

In this cross-sectional study of a random population of healthy young adults, aged 18–25 years, we found a positive, systematic and equal effect of physical exercise on the BMD at all DXA-scan sites. We further observed an association between the BMD at all sites and vitamin D status. The BMD was significantly higher, on average 0.10 (0.03, 0.17) g/cm^2^ (adjusted for sex, exercise levels, and site) for the group with a sufficient vitamin D concentration compared to the participants with vitamin D insufficiency. For sexes compared, men had higher BMD at femoral necks and total hips, while BMD was equal for sexes at lumbar spine.

Our above results align with the current studies performed in young men and women. Young men at similar geographical latitudes with a s-25[OH]D of <44 nmol/L were found to have a lower BMD at the total hip and lumbar spine compared with those participants with a s-25[OH]D of >44 nmol/L (Valimaki et al. 2004). Nevertheless, this difference in young men could be partially explained by the use of different cut-off values. For children (age range 7–19 years) s-25[OH]D is a strong predictor for BMD and levels of s-25[OH]D (Pekkinen et al. 2012). Vitamin D effects bone elongation, calcification and remodeling (Lui et al. 2010; Boyan et al. 2003; Dusso et al. 2005). The two groups did not differ significantly in height, as presented in Table 1. Thus, this rendered the possible impact of vitamin D on bone elongation, represented by height, to be likely insignificant. The other manifestations of calcification and remodeling are hard to distinguish from one another. However, our BMD data at total hip and spine indicate that exercise may serve to increase the BMD, regardless of the vitamin D level and sex. The male participants had a higher BMD at the femoral neck and total hip, bilaterally, but not at the lumbar spine. These results are in line with the findings reported by other investigators (Kelly et al. 1990; Valero et al. 2005; Looker et al. 2012), where the indifference of the BMD to the variable of sex at the lumbar spine is caused by the longer and wider vertebra found in men (Kelly et al. 1990; Looker et al. 2012).

In our study, we found that exercise was positively associated with the BMD at all sites. This observation was independent of the gender and vitamin D status of the participants. It is well-known, however, that exercise is associated with a higher BMD, especially in high-impact training (Eleftheriou et al. 2012). The positive association between exercise and intestinal calcium uptake (Christakos et al. 2011) could explain some of the positive associations between BMD and exercise.

The positive effect of BMD from exercise has been reported for decades, but the role of sex and exercise on BMD remains a subject of investigation. In pre-pubertal children who are still accruing bone mass, there is a sex difference in the effect of exercise on the BMD for the total hip and femoral neck (Cardadeiro et al. 2012; Kriemler et al. 2008). In our study of young adolescents, we found that exercise was positively associated with the BMD at all sites, independent of sex and for all levels of exercise.

Notably, Vitamin D supplementation has a known positive effect on muscle strength, however, the authors did not investigate the influence of baseline s-25[OH]D (Tomlinson et al. 2014), and physical fitness has been shown to be associated with the BMD (Schwarz et al. 2014). Hypothetically, an increase in muscle strength caused by vitamin D could translate into a different stimulus to the bones of the two groups. Whether the two groups had different muscle strengths is unknown, and the groups could theoretically be different in the specific loads applied to their bones and, thus, have differential stimulation of their bones. Our data do not favor a higher effect of training in the group with sufficient vitamin D status, although this could be due to limitations in the data given that exercise was measured as a discrete variable. Thus, exercise as a discrete variable is a limitation of the study; if exercise had been measured as a continuous variable, it would have been possible to provide a more precise estimation of the exercise effect.

There are many limitations of a cross-sectional study. In our study, the selection of a population of healthy young persons with recreational physical exercise was conducted based on educational status. On the other hand, our inclusion of multiple education levels can be viewed as strength and could make our results comparable to a contemporary background population. However, the youth outside the educational system were not included. We could not include the number of participants as we aimed for with the power calculation; this was due to eligible subjects, with sufficient s-25[OH]D in the screening process; this should be considered a weakness. We have not controlled for recent holidays in sunny climate, which is known to affect s-25[OH]D (Petersen et al. 2014) and should be considered a limitation.

Furthermore, the utilized cross-sectional design provides us with a snapshot of the exercise habits and the bone status of the participants, which may reflect the effects of habitual loading patterns over long periods. This is in contrast to interventional studies that only investigate the immediate effect of exercise. Nevertheless, the cross-sectional study design makes it impossible to differentiate a true cause and effect relationship.

## Conclusion

We conclude that the BMD in healthy young adults is influenced by physical exercise, independent of sex and vitamin D status. The group with sufficient vitamin D levels had a systematically higher BMD for all levels of physical exercise; however, for both the vitamin D sufficient and insufficient groups, physical exercise was equally associated with an increase in the BMD. Lastly, for both sexes, the BMD was equally associated with physical exercise.

## Abbreviations

1,25[OH]D: 1,25-dihydroxyvitamin D
25[OH]D: 25-hydroxyvitamin D
BMI: Body mass index
Cl: Confidence limits
CV: Coefficient of variation
DXA: Dual-energy X-ray absorptiometry
PBM: Peak bone mass
PTH: Parathyroid hormone
s-: Serum
